# Selective Inflammatory Pain Insensitivity in the African Naked Mole-Rat (Heterocephalus glaber)

**DOI:** 10.1371/journal.pbio.0060013

**Published:** 2008-01-29

**Authors:** Thomas J Park, Ying Lu, René Jüttner, Ewan St. J Smith, Jing Hu, Antje Brand, Christiane Wetzel, Nevena Milenkovic, Bettina Erdmann, Paul A Heppenstall, Charles E Laurito, Steven P Wilson, Gary R Lewin

**Affiliations:** 1 Laboratory of Integrative Neuroscience, Department of Biological Sciences, University of Illinois at Chicago, Chicago, Illinois, United States of America; 2 Department of Anesthesiology, University of Illinois at Chicago, Chicago, Illinois, United States of America; 3 Max-Delbrück Center for Molecular Medicine, Berlin, Germany; 4 Department of Electron Microscopy, Max-Delbrück Center for Molecular Medicine, Berlin, Germany; 5 Klinik für Anaesthesiologie und Operative Intensivmedizin, Charité Universitätsmedizin Berlin, Campus Benjamin Franklin, Berlin, Germany; 6 Department of Pharmacology, Physiology, and Neuroscience, University of South Carolina School of Medicine, Columbia, South Carolina, United States of America; University of California San Francisco, United States of America

## Abstract

In all mammals, tissue inflammation leads to pain and behavioral sensitization to thermal and mechanical stimuli called hyperalgesia. We studied pain mechanisms in the African naked mole-rat, an unusual rodent species that lacks pain-related neuropeptides (e.g., substance P) in cutaneous sensory fibers. Naked mole-rats show a unique and remarkable lack of pain-related behaviors to two potent algogens, acid and capsaicin. Furthermore, when exposed to inflammatory insults or known mediators, naked mole-rats do not display thermal hyperalgesia. In contrast, naked mole-rats do display nocifensive behaviors in the formalin test and show mechanical hyperalgesia after inflammation. Using electrophysiology, we showed that primary afferent nociceptors in naked mole-rats are insensitive to acid stimuli, consistent with the animal's lack of acid-induced behavior. Acid transduction by sensory neurons is observed in birds, amphibians, and fish, which suggests that this tranduction mechanism has been selectively disabled in the naked mole-rat in the course of its evolution. In contrast, nociceptors do respond vigorously to capsaicin, and we also show that sensory neurons express a transient receptor potential vanilloid channel-1 ion channel that is capsaicin sensitive. Nevertheless, the activation of capsaicin-sensitive sensory neurons in naked mole-rats does not produce pain-related behavior. We show that capsaicin-sensitive nociceptors in the naked mole-rat are functionally connected to superficial dorsal horn neurons as in mice. However, the same nociceptors are also functionally connected to deep dorsal horn neurons, a connectivity that is rare in mice. The pain biology of the naked mole-rat is unique among mammals, thus the study of pain mechanisms in this unusual species can provide major insights into what constitutes “normal” mammalian nociception.

## Author Summary

Chemicals such as capsaicin and acid are considered noxious because they cause irritation and pain when applied to the skin. Acid is, for example, a very noxious stimulus and can cause intense pain. Indeed, acid is both noxious and painful to all animals including amphibians and fish. Here we describe a member of the rodent family, the African naked mole-rat (Heterocephalus glaber), that is behaviorally completely oblivious to capsaicin and acid. Tissue injury and inflammation increase sensitivity to normally non painful stimuli, a phenomenon called hyperalgesia. Here we show that the naked mole-rat does not experience hyperalgesia to painful thermal stimuli after inflammation. To our knowledge, no other mammal has so far been described that is selectively insensitive to chemical pain or that lacks thermal hyperalgesia. Naked mole-rats live in very large subterranean social groups and are remarkably tolerant to low-oxygen and high–carbon dioxide conditions. We hypothesize that naked mole-rats are selectively pain insensitive partly because of selection pressure arising from the extremity of their normal habitat.

## Abstract 3

Naked but far from vulnerable, the African naked mole-rat is an unusual mammal that is unique because it is impervious to painful chemicals that cause severe pain in all other species studied.

## Introduction

The neurobiology of pain is of fundamental interest. Pain is usually experienced in two main contexts. First acutely, for example, pin-prick or contact with a hot object prompts a rapid withdrawal from the noxious stimulus. The second, more clinically relevant, context for pain is that following tissue injury or inflammation. Long lasting inflammation is often associated with ongoing pain and enhanced sensitivity to stimuli that are normally only mildly unpleasant, so-called hyperalgesia. Inflammatory pain following tissue damage usually lasts for many hours or even days. The development of inflammatory pain is a complex process involving events at the site of injury, in primary sensory neurons and the central nervous system (CNS) [1–3]. Tissue injury triggers the release of inflammatory mediators that can stimulate and sensitize specialized sensory neurons, called nociceptors, that detect stimuli harmful to the organism [1,4]. The activation of nociceptors in turn drives synaptic circuits in the dorsal horn of the spinal cord that exhibit activity-dependent plasticity. It is widely accepted that this nociceptor-driven plasticity can produce a central sensitization state required for the maintenance of hyperalgesia [2,3].

The physiology and organization of pain pathways appear to be highly conserved across all vertebrate taxa. Support for this notion comes from detailed physiological studies showing that the properties of nociceptors vary little when compared in many diverse vertebrate species, including frogs, birds, cats, primates, and even some fish [5–8]. All vertebrate species that have nociceptors exhibit pain-related behaviors when confronted with noxious stimuli that would be painful to humans.

Nociceptors also detect environmental irritants; one well-studied example is capsaicin, the active ingredient of chili peppers. Capsaicin produces intense burning pain in humans and characteristic pain behaviors in most vertebrates studied with, the notable exception of birds [9–11]. The capsaicin receptor is a nonselective cation channel called transient receptor potential vanilloid channel-1 (TRPV1), which can also be activated by noxious heat and protons [11,12]. Interestingly, avian TRPV1 receptors do not respond to capsaicin, and this is the basis for the fact that chili peppers are eaten by birds that are also effective seed dispersers. In contrast, small mammals avoid the chili fruit in the wild and are poor seed dispersers [13]. The capsaicin insensitivity of the avian TRPV1 was used to determine the amino acid residues that are required for channel activation by capsaicin [10]. Here we provide a description of a mammal, the African naked mole-rat (Heterocephalus glaber), that exhibits complete behavioral insensitivity to capsaicin.

Naked mole-rats belong to the family Bathyergidae. They are found in abundance in central East Africa, where they live in large subterranean colonies of up to 300 individuals [14]. This species has been of interest to ecologists for some time because of their insect-like social structure [15]. Naked mole-rats are unusual, even among other subterranean and mole-rat species, in that they are the only known cold-blooded mammal [16], and they are extremely long lived (lifetimes in excess of 25 y) [17]. We recently reported that African naked mole-rats (H. glaber) are also unique among mammals in that they naturally lack substance P (SP) and calcitonin gene-related peptide (CGRP) in the skin innervation [18]. These sensory neuropeptides are usually associated with nociceptive primary afferent fibers, and in the absence of either of these peptides, pain-related behaviors are reduced [19,20]. These findings prompted us to undertake a systematic anatomical, behavioral, and physiological analysis of nociception in naked mole-rats.

We show that naked mole-rats, in addition to being insensitive to the effects of capsaicin, are the only known vertebrate without behavioral sensitivity to acid and, in addition, display no thermal hyperalgesia following peripheral inflammation. Naked mole-rat nociceptors completely lack the ability to detect acid, a transduction capability that is found in all vertebrates studied to date including mammals, birds, and fish [8,21–23]. Surprisingly, the behavioral insensitivity to capsaicin observed in naked mole-rats is, unlike in birds, not due to nociceptor insensitivity to capsaicin. Nociceptors in naked mole-rats respond to capsaicin, but the functional connectivity of capsaicin-sensitive sensory fibers in the spinal cord dorsal horn is substantially different from that found in other rodent species. Thus we show that specific aspects of nociceptor physiology and connectivity are unique in the naked mole-rat. We suggest that the extreme native habitat of a naked mole-rat ancestor may have triggered the evolution of this unusual nociceptive system.

## Results

### Anatomy and Physiology of Sensory Neurons in the Naked Mole-Rat

**Figure 1:**
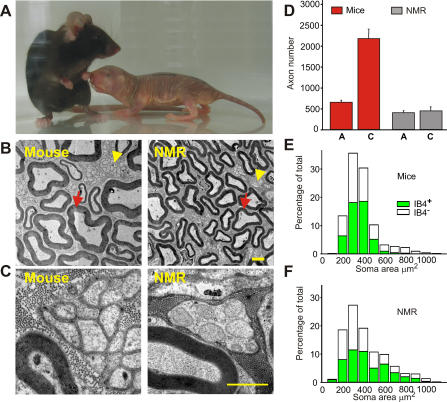
Comparison of Myelinated and Unmyelinated Axons in the Saphenous Nerve of Adult Laboratory Mice and 1-y-Old Naked Mole-Rats (A) Photograph showing the similar overall size of the animals as well as the similar size of the hind feet, which are innervated by the saphenous nerve. (B) Representative electron micrographs showing unmyelinated axons within Remak bundles (yellow arrows) and myelinated axons (red arrows) in saphenous nerves from the mouse and naked mole-rat (NMR). Scale bar = 2.0 μm. (C) At higher magnification, single unmyelinated fibers can be distinguished within the Remak bundles. Scale bar = 2.0 μm. (D) Quantification of myelinated and unmyelinated axon numbers in mouse (*n* = 6) and naked mole-rat (*n* = 4) saphenous nerves. Note that here and in subsequent figures, red bars denote data from mouse and gray bars indicate data from naked mole-rats. (E and F) Proportion of cultured sensory cells that label with IB4 in mice (E) and naked mole-rats (F). Distributions are based on soma area, and C-cells have soma areas less than 600 μm^2^.

We studied the saphenous nerve, which innervates the skin of the medial hind limb and paw, because primary afferents in this nerve have been well characterized anatomically and physiologically in many species [23–25]. We made an electron microscopic analysis of the saphenous nerve in the naked mole-rat to determine the number and morphology of myelinated A-fiber axons and unmyelinated C-fiber axons. We used laboratory mice as a comparison species, because their feet, as well as overall body, are of comparable size (Figure 1A). The ultrastructure of both C-fiber axons in Remak bundles and myelinated fibers in the naked mole-rat is qualitatively similar to that in the mouse (Figure 1B and 1C). We counted the number of A- and C-fiber axons and found that the saphenous nerve of naked mole-rats contains about 30% fewer myelinated axons than that of the mouse. The myelinated axons had a smaller diameter in the naked mole-rat compared to the mouse (Figure S1), and this was reflected in slower conduction velocities (Table 1). It has often been observed that cutaneous nerves of rodents, primates, and humans contain many more unmyelinated fibers than they do myelinated fibers. For example, in rats, mice, and humans, cutaneous nerves contain four times as many C-fiber than A-fiber axons [25–27]. We found that in the naked mole-rat, saphenous nerve unmyelinated C-fibers were scarce compared with other species. The mean ratio of C-fiber axons to A-fiber axons in this species is 1.1 compared to 3.8 in the mouse (Figure 1D).

**Table 1:**
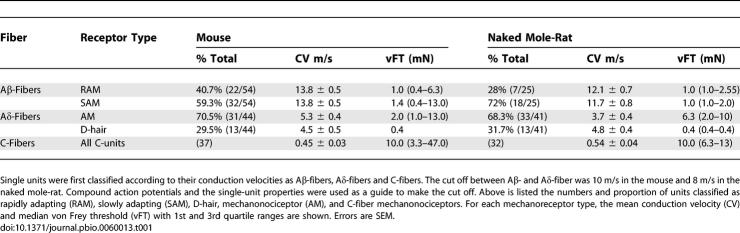
The Physiological Properties of Afferents Recorded from Naked Mole-Rats Compared to Those in Mice

In rats and mice, nociceptors in the dorsal root ganglia (DRG) can be divided into two major neurochemical classes: those that express the pain related neuropeptides CGRP and SP and non-peptidergic cells defined by their cell-surface binding of the isolectin B4 (IB4) [28,29]. The paucity of sensory neuropeptides in this species led us to ask whether the entire nociceptor population is IB4-positive in the naked mole-rat. We made primary cultures of DRG neurons from adult naked mole-rats and mice and used a fluorescently labeled IB4 to stain small-diameter neurons [30]. We plotted the cell size distribution of IB4-positive and -negative cells, and as expected, we noted that there is large relative reduction in the incidence of small sensory neurons in the naked mole-rat compared to mice (Figure 1E and 1F). IB4 positive cells make up around half of the small-diameter neurons in the mouse, and this was also the case for naked mole-rat sensory cells (Figure 1E and 1F). It thus appears that the lack of neuropeptide expression in the naked mole-rat DRG is not due to a complete loss or conversion of this cell type into nonpeptidergic IB4-positive cells. We also noted that many medium- and large-diameter naked mole-rat sensory neurons bind IB4, something not observed in rats or mice [30,31]. It remains to be determined if these larger IB4-positive cells are nociceptors.

We next carried out a detailed electrophysiological study of the receptive properties of cutaneous afferents in the naked mole-rat. We used an in vitro skin nerve preparation [24] to make recordings from single A- and C-fiber afferents in the saphenous nerve. Recordings were made from a total of 91 single A-fibers and 32 C-fibers in 17 animals ranging from 1–5 y of age. We found that naked mole-rat mechanoreceptors and nociceptors could be classified in broadly the same way as in the mouse (Table 1). Two major groups of C-fiber nociceptors can be defined on the basis of their responses to noxious thermal and mechanical stimuli. Most C-fibers respond to noxious heat as well as mechanical stimulation and are termed polymodal or C-mechanoheat fibers (C_MH_), whereas the remaining C-fibers are heat insensitive and classified as C-mechanonociceptors (C_M_) [6].

**Figure 2:**
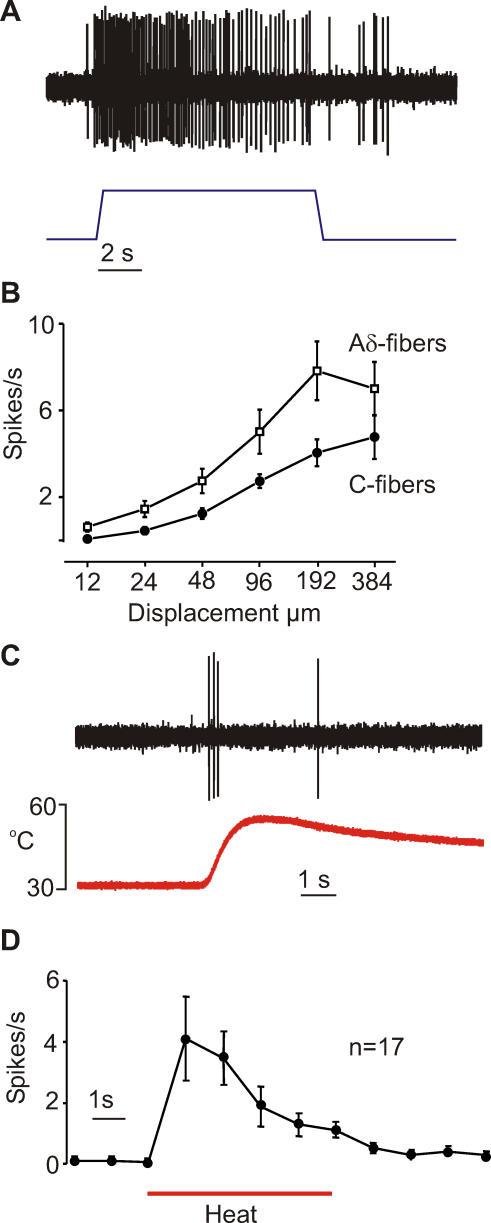
Recordings from Naked Mole-Rat Single Afferent Fibers in the Saphenous Skin Nerve Preparation (A) Example of a recording made from a single C-fiber afferent and its response to mechanical stimulation, indentation of 192 μm. (B) Stimulus-response relations of mechanosensitive Aδ and C-fiber afferents for a standard series of ascending displacement stimuli applied to the receptive fields. (C) Example of a polymodal C-fiber (C_MH_) response to application of heated bath solution onto its receptive field. (D) Averaged spike rates for 17 C_MH_ fibers before and during the heating stimulus. An additional 13 C_M_ fibers did not respond to heating.

We characterized responses to mechanical stimuli by presenting a series of standard indentation stimuli ranging from 12–384 μm. The example trace in Figure 2A shows the response of a single C-fiber to an indentation of 192 μm. Stimulus-response functions were calculated for each neuron, and the mean functions for all nociceptors are plotted in Figure 2B. These functions are very similar to those previously reported for mice using the same methodology [24,32]. To test heat sensitivity, we applied heated bath solution to a unit's receptive field. The trace in Figure 2C is from a C-fiber that fired action potentials in response to heating. We found that 57% of single C-fibers (17/30 fibers) responded to heat and these were classified as C_MH_. The average response rates for these cells before and after heating are plotted in Figure 2D, and the remaining C-fibers were classified as C_M_.

Many of the afferent axons with conduction velocities in the Aδ-fiber range had physiological properties characteristic of A-fiber mechanonociceptors (AM) including high von Frey thresholds (Table 1). The stimulus-response function of AM fibers in naked mole-rats is very similar to that described for the same fibers in mice. Characteristically this class of nociceptive fiber displays higher firing rates to intense mechanical stimuli than do C-fibers (Figure 2B).

### Pain Behavior in the Naked Mole-Rat

**Figure 3:**
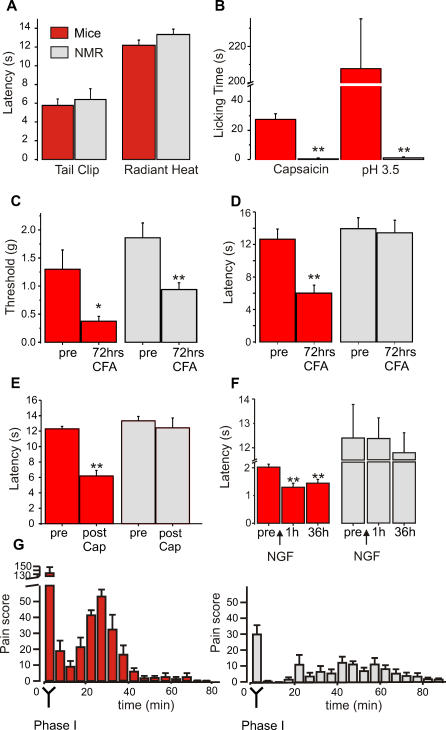
Behavioral Responses to Acute Noxious Stimuli and Inflammatory Pain Stimuli in Mice and Naked Mole-Rats (A) Response latency to tail clip (450 g pressure, *n* = 25 mice, 25 naked mole-rats [NMR]), and paw withdrawal latency to radiant heat (*n* = 12 mice, 12 NMR). (B) Behavioral responses to foot pad injection of capsaicin (*n* = 10 mice, 10 NMR) and acidic saline solution (pH 3.25, *n* = 5 mice, 5 NMR), measured as time spent licking the affected paw. Note virtually no behavioral response in terms of paw licking was found for either stimulus in naked mole-rats. (C) Paw withdrawal thresholds to mechanical stimulation with von Frey hairs before and during inflammation from an injection of CFA; both mice and naked mole-rats show significant sensitization during inflammation (*n* = 10 mice, 10 NMR). (D) Paw withdrawal to radiant heat before and 72 h after injection of CFA; mice show a significant sensitization but naked mole-rats show no sensitization (*n* = 6 mice, 6 NMR). (E) Paw withdrawal to radiant heat before and after topical application of 1 mM capsaicin (Cap) ( = 6 mice, 12 NMR); mice show a significant sensitization (shortened latency) after capsaicin, naked mole-rats show no sensitization. (F) Paw withdrawal to radiant heat before and after a systemic injection of nerve growth factor (NGF 1μg/g); mice show a significant sensitization (shortened latency) at 1 h, and this is maintained at 36 h post injection; naked mole-rats show no sensitization at either time point. For panels A-F *p < 0.05; **p < 0.01 Student's *t*-test. (G) Frequency of pain behaviors to injection of 1% formalin; pain behaviors are significantly attenuated in naked mole-rats compared to mice in both phase I (0–5 min, *p* < 0.0001) and phase II (>5 min, *p* < .01) of the formalin test (*n* = 6 mice, 6 NMR).

We tested naked mole-rats in standard behavioral models of acute pain including tests for mechanical (pinch), thermal, and chemical pain. We found that for noxious pinch and heat, the mole-rats responded similarly to mice. Both species showed similar latencies to bite at a clip applied to the base of the tail (Figure 3A); the pressure applied was identical for mice and naked mole-rats (450g). Likewise, both species show similar latencies for hind limb withdrawal to radiant heat; the same heat ramp was used for each species (Figure 3A). In contrast to the results using mechanical and thermal stimuli, there was a striking difference in responses to strong chemical irritants known to excite nociceptors. Indeed the two chemicals used—capsaicin and low-pH saline solution—normally evoke very intense pain in humans and other animals [9,33,34]. Injection of either irritant into the skin rapidly evoked intense licking and guarding behaviors in mice. In contrast, naked mole-rats showed virtually no behavioral response to either noxious irritant (Figure 3B).

We went on to examine the behavior of naked mole-rats in a number of inflammatory pain models. Injection of formalin into the paw (formalin test) generates a stereotyped, biphasic period of pain behaviors lasting about 1 h [35]. Within the first 5 min (phase I), pain behavior is primarily driven by C-fiber activity. This is followed by a short reduction in behavior and then a longer lasting increase (phase II) that is thought to be due at least in part to central sensitization [35]. During phase I, naked mole-rats showed a reduced frequency of pain behaviors compared to mice (total pain score about 20% of that in mouse) (Figure 3G). A similar reduced pain score was also observed in phase II compared to mice, although a low frequency of pain-related behaviors tended to persist longer in naked mole-rats than in mice. The observation that naked mole-rats respond to formalin with licking and guarding behaviors (albeit reduced in frequency) is important, because it indicates that these behaviors are part of the naked mole-rats' behavioral repertoire. Therefore, a behavioral deficit cannot explain the complete lack of pain behavior observed after paw injection of capsaicin and acid.

Hyperalgesia, both to thermal and mechanical stimuli, are hallmarks of inflammatory pain. We next asked whether the mechanisms of hyperalgesia were also altered in the naked mole-rat compared to other species. Injection of complete Freund's adjuvant into the paw (CFA model) generates local inflammation that lasts for days [36]. Within hours of CFA injection, mice displayed a heightened sensitivity to both noxious mechanical and thermal stimuli (mechanical and thermal hyperalgesia) that peaked 24–72 h after the injection (Figure 3C and 3D). Naked mole-rats displayed a different pattern in response to CFA: although they showed mechanical hyperalgesia similar in magnitude to that of mice (Figure 3C), they showed no sign of thermal hyperalgesia (Figure 3D). Despite this lack of thermal hyperalgesia, paw edema was comparable to that seen in mice, with an increase in paw diameter of 22.4% for naked mole-rats and 19.4% for mice.

Activation of chemosensitive nociceptors can also produce long-lasting thermal hyperalgesia, independent of inflammation. Both topical capsaicin and the systemic or local injection of nerve growth factor (NGF) induce thermal hyperalgesia in rodents and humans [33,36–39], which is dependent on the presence of TRPV1 [40,41]. Consistent with previous reports, both capsaicin and NGF produced a profound and long-lasting thermal hyperalgesia in mice (Figure 3E and 3F). In contrast, naked mole-rats did not show thermal hyperalgesia to capsaicin (Figure 3E), which is consistent with the lack of acute sensitivity to this compound when injected (Figure 3B). But NGF also failed to produce heat hyperalgesia both acutely (<4 h) and chronically (>24h) (Figure 3F), which was surprising because the acute effects of NGF in rodents are due to a direct sensitization of primary nociceptors to heat and not to direct excitation of cutaneous nociceptors [36,42]. The lack of NGF-induced thermal hyperalgesia cannot be explained by a lack of functional NGF receptors, because we were able to observe a robust stimulation of neurite-growth in cultured small and medium sensory neurons that were also stained positive for the NGF receptor TrkA (Figure S2).

**Figure 4:**
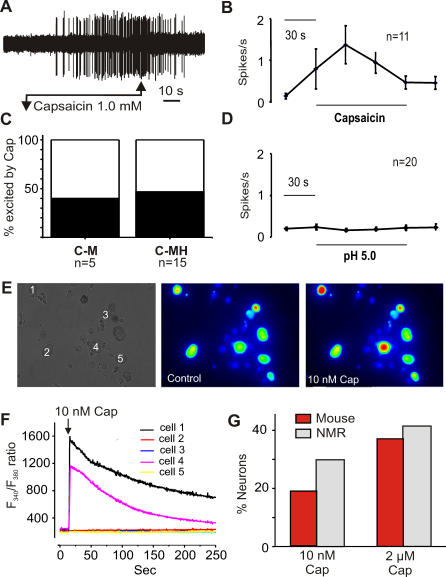
Capsaicin Sensitivity of Single Peripheral Nerve Fibers in the Saphenous Skin Nerve Preparation and for Sensory Neurons in Culture (A) An example of a polymodal C-fiber (C_MH_) response to local application of a 1 mM capsaicin solution to its receptive field. (B) The average firing rate of 11 capsaicin-sensitive C-fibers during the 2 min application. (C) The proportion of C_M_ and C_MH_ fibers showing an excitatory response to capsaicin. (D) The rate of firing of C_MH_ fibers during application of pH 5.0 saline solution to the receptive field; no increase in firing rate was found in any of the tested fibers during acid application (*n* = 20). (E) Calcium imaging experiments were performed to measure the intracellular calcium changes in response to capsaicin applied to sensory neurons in culture. A typical experiment is shown, where a group of cells from naked mole-rat were monitored, the phase contrast image is shown on the left. An image of the Fura-2 fluorescence signal measured when excited at 340 nm is shown for the same cells under resting conditions and in the presence (right) and absence (middle) of 10 nM capsaicin. (F) Two of the five cells shown displayed a large increase in intracellular calcium upon application of capsaicin (10 nM) as shown in the F340/F380 ratio. (G) Quantification of the proportion of imaged sensory neurons showing an increase in intracellular calcium after application of 10 nM or 2 μM capsaicin to naked mole-rat or mouse sensory neurons (*n* = 69 and *n* = 16). No significant differences were observed in the proportion of capsaicin-sensitive neurons between mouse and naked mole-rat.

We speculated that the reduced pain behavior in the naked mole-rat could simply be explained by a lack of functional TRPV1 ion channels. Accordingly, we used the skin nerve preparation to determine the chemical sensitivity of naked mole-rat primary afferent nociceptors. Considering the naked mole-rats' behavioral insensitivity to capsaicin, we were surprised to find that approximately 40% of C_MH_ and C_M_ fibers respond robustly to 1 mM capsaicin (Figure 4A–4C), the same concentration of capsaicin used in the behavioral experiments. The rates of nociceptor firing following excitation by capsaicin were in the range of those evoked by noxious thermal and mechanical stimuli (Figure 2). Nevertheless the concentrations of capsaicin that we used in the skin nerve preparation were high, considering the low nanomolar affinity that TRPV1 has for capsaicin [1,11]. We thus used cultures of naked mole-rat DRG neurons and applied low concentrations of capsaicin onto single cells and measured receptor activation using fura-2–based calcium imaging (Figure 4E and 4F). The results of these experiments indicated that the incidence and magnitude of the capsaicin responses to both low (10 nM) and high (2 μM) capsaicin concentrations are equivalent to that found in the mouse (Figure 4G).

It has been known for some time that a large proportion of C_MH_ fibers in rats and mice also show robust sustained responses to acidic pH [43,44]. We tested 20 naked mole-rat C-fibers for proton sensitivity and found that neither C_MH_ nor C_M_ fibers were excited by protons, because low-pH solutions ranging from pH 6.0 to pH 5.0 had no excitatory effect (Figure 4D). In contrast, it has been reported that ∼40% of C_MH_ fibers in the mouse can be robustly excited by the same stimuli [43]. The lack of proton sensitivity of single nociceptors can therefore completely explain the naked mole-rats behavioral insensitivity to acid (Figure 2B).

### Central Connectivity of C-Fibers Is Altered in the Naked Mole-Rat

**Figure 5:**
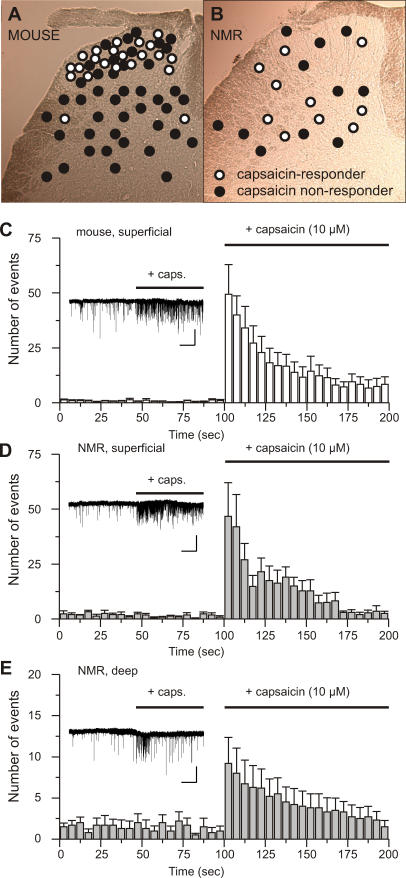
Patch Clamp Recordings of Spinal Cells in Superficial and Deep Lamina of the Dorsal Horn with Bath Application of Capsaicin (10 μM) (A and B) Locations of cells in mouse (A) and naked mole-rat (B) spinal slices. White dots indicate cells that responded to capsaicin; black dots indicate cells that did not respond to capsaicin. Scale bar = 100 μm. (C) Mean number of mEPSCs recorded from all superficial mouse cells that responded to capsaicin. The histogram shows 100 s before and 100 s after bath application of capsaicin. The inset trace shows a typical recording from one mouse cell. Scale bars, 10 s, 10 pA. (D) Mean number of mEPSCs from four naked mole-rat cells that were located in superficial dorsal horn and responded to capsaicin. (E) Mean number of mEPSCs from six naked mole-rat cells that were located in deep dorsal horn and responded to capsaicin. Miniature EPSCs were recorded in the presence of strychnine (1 μM), picrotoxin (100 μM), and APV (100 μM) to block glycinergic and GABAergic input as well as NMDA receptor-mediated currents. TTX (1 μM) was used to block action-potential dependent neurotransmitter release. Bin size for (C–E), 5 s.

Our finding that naked mole-rats are behaviorally insensitive to capsaicin but have sensory neurons that respond normally to this irritant raised the following question: do capsaicin-sensitive C-fibers make functional connections in the spinal cord? We addressed this question by using whole-cell patch clamp recordings from dorsal horn neurons in transverse slices of spinal cord taken from juvenile mice (post-natal day 10–14 [p10–14]) and naked mole-rats. The slices were perfused with tetrodotoxin (TTX) to block action potential propagation and a cocktail of antagonists to isolate glutamatergic connections (see Materials and Methods). Once a whole-cell recording was achieved, miniature excitatory post-synaptic currents (mEPSCs) were recorded. The slice was superfused with 10 μM capsaicin and, if the recorded cell has direct monosynaptic connections with capsaicin-sensitive nociceptors, an increase in mEPSC frequency should be observed [45,46]. We found that 57% (16/28) of cells recorded in the mouse superficial dorsal horn responded with a substantial increase in mEPSC frequency after capsaicin application (Figure 5A and 5C). However, only two out of 30 cells recorded in deeper lamina in the mouse (6.6%) responded with increased mEPSC frequency to capsaicin superperfusion (Figure 5A). We made whole-cell recordings from superficially located neurons in slices from naked mole-rats, and the properties of the mEPSCs (amplitude, decay time, frequency) were essentially identical to those recorded in the mouse (Table S1). Also similar to the mouse, capsaicin in the naked mole-rat produced a substantial increase in mEPSC frequency in 50% (4/8) of the cells recorded in the superficial dorsal horn (Figure 5B and 5D). However, when we recorded cells located in the deep dorsal horn, we found that 46% (6/13) of the cells tested exhibited a pronounced increase in mEPSC frequency after superfusion with capsaicin (Figure 5B and 5E). The difference between the proportion of capsaicin-responding neurons in the mouse deep dorsal horn (6.6%) compared to the naked mole-rat (46%) was statistically significant (Chi squared test *p* < 0.01). Thus, TRPV1-responsive sensory fibers are synaptically connected to both superficial and deep dorsal horn neurons in the naked mole-rat. This direct connectivity between TRPV1-bearing fibers and deep dorsal horn neurons is much less frequent in the mouse than in the naked mole-rat spinal cord.

**Figure 6:**
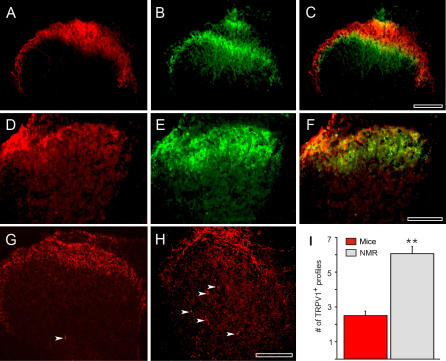
Comparison of TRPV1 Staining in the Spinal Cord of the Mouse and Naked Mole-Rat Sections were stained with a primary anti-rat TRPV1 antibody that was detected with a secondary antibody conjugated to Cy3 (red), sections were co-stained with IB4 conjugated to Alexa-488 (green). (A–C) TRPV1 and IB4 staining is localized to the superficial laminae of the dorsal horn in the mouse. (D–F) Similar staining is observed in the naked mole-rat, although a thicker lamina II gives rise to increased IB4 staining. (G) Very little TRPV1 deep staining is observed in the mouse. (H) Deep TRPV1 staining is frequently observed in the naked mole-rat; white arrows highlight TRPV1 positive profiles in deeper laminae. (I) Quantification of TRPV1-positive profiles in deep dorsal horn of mouse and naked mole-rat. Scale bars for (A–H) are 100 μm.

We went on to examine the distribution of TRPV1-positive fibers and varicosities in the spinal cord using immunocytochemistry. TRPV1-positive fibers in the mouse are predominantly found in the superficial dorsal horn [12]. We thus compared the distribution of TRPV1-positive fibers and varicosities in the naked mole-rat and the mouse (Figure 6). Double staining experiments revealed that in the mouse, TRPV1-positive profiles are more superficially located in laminas I and II outer, compared to IB4-positive fibers, which extend into lamina II inner (Figure 6A–6C). This distinction was less clear for the naked mole-rat, where many TRPV1 fibers were located deep in lamina II and overlapping with the IB4-positive fibers (Figure 6D–6F). In the deep dorsal horn, occasional TRPV1-positive fibers could be identified in the mouse cord (Figure 6G), but in the naked mole-rat deep dorsal horn, such TRPV1-positive profiles were more common (Figure 6H). A quantitative analysis of the incidence of deep TRPV1-positive profiles was carried out (30 sections from three mice compared with 33 sections from three naked mole-rats), and the mean incidence of such profiles was found to be more than twice as high in the naked mole-rat compared with in the mouse (Figure 6I).

The unusual connectivity of capsaicin-sensitive C-fibers in the naked mole-rat led us to ask whether the organization of C-fiber–driven reflexes may also be altered in the naked mole-rat. We measured ventral root reflexes using a hemisected in vitro spinal cord preparation. We find that activation of C-fibers leads to a very long-lasting activation of flexor motoneurons reflected in a long-lasting ventral root potential (>20 s) to single shock stimulation of C-fibers in the attached dorsal root [47]. In naked mole-rats, the time course of the ventral root potential was much shorter than that found in neonatal mice (Figure S3).

### Reintroduction of SP Can “Rescue” Pain-Related Behavior in Naked Mole-Rats

**Figure 7:**
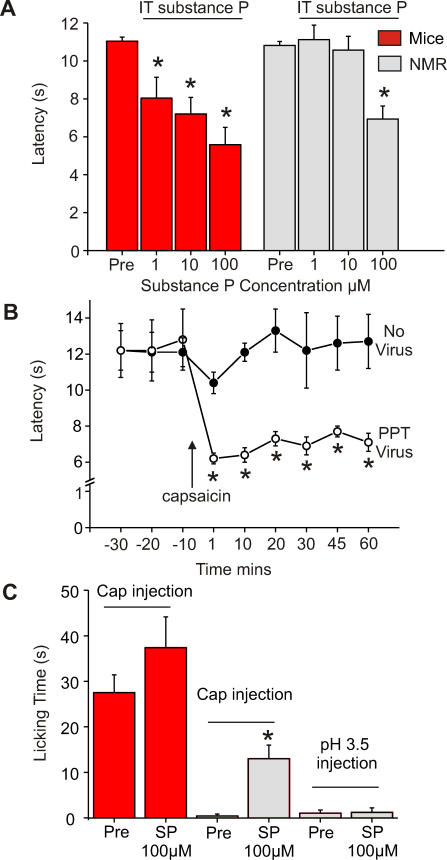
Hyperalgesia and Pain following Central Administration of SP (A) Paw withdrawal to radiant heat measured before and after administration of SP peptide to the lumbar spinal cord via an intrathecal cannula in lightly anesthetized animals (IT SP). Note that very low doses of SP lead to thermal hyperalgesia in mice (1 μM). Naked mole-rats also display hyperalgesia following intrathecal SP injection albeit only at higher doses (100 μM, *n* = 6 animals per dose). (B) Paw withdrawal to radiant heat was measured before and 1 wk after infection of one paw with transgenic herpes virus carrying the preprotachykinin gene (*n* = 4 naked mole-rats [NMR]). The virus treatment alone did not alter thermal thresholds (first three data points), but in contrast to naive animals, topical capsaicin leads to thermal hyperalgesia (shortened latencies) in the virus-treated paw. (C) Pain behavior following injection of capsaicin but not acid is observed following intrathecal administration of SP. Mice and naked mole-rats were lightly anesthetized via inhalation and given an intrathecal injection of 100 μM SP. After recovery from the anesthetic (∼5–10 min) the animals received a paw injection of capsaicin (*n* = 6 mice, 5 NMR) or acidic (pH 3.5) saline solution (*n* = 6 NMR) into one foot pad, and paw licking time was measured. Note that SP slightly increased licking behaviors in mice compared to capsaicin injection alone (Pre). Naked mole-rats showed significant licking behavior after intrathecal SP which was virtually absent in untreated animals. No change in the behavioral response to a pH 3.5 solution was observed.

In addition to displaying a highly unusual connectivity of nociceptive sensory neurons, the naked mole-rat also lacks the sensory neuropeptides SP and CGRP in the skin [18]. We asked whether reintroduction of SP into the naked mole-rat might be capable of “normalizing” pain behavior in this species. We measured noxious heat withdrawal latencies continuously before and after intrathecal infusion of SP (1–100 μM) in lightly anesthetized animals. Thermal hyperalgesia was observed within minutes after infusion of 1μM SP in mice. Naked mole-rats also displayed thermal hyperalgesia, but only at higher does (100 μM) (Figure 7A). In a second series of experiments, we used a neurotropic herpes virus engineered to express the *preprotachykinin* gene (*PPT*) to infect naked mole-rat DRG neurons innervating one paw. We tested the animals 1 wk after infection and found that thermal nociceptive latencies were unchanged (Figure 7B, first three data points). However, the naked mole-rats now displayed a robust thermal hyperalgesia when we applied topical capsaicin to the virus-treated paw at a concentration that produces reliable heat hyperalgesia in mice (1 mM).

Finally, we asked if introduction of SP could also enable behavioral sensitivity to acute application of capsaicin and acid. We found that 10 min after intrathecal administration of SP (100μM), an injection of capsaicin into the skin of the paw now caused naked mole-rats to display robust guarding and licking behaviors (Figure 7C). This result shows that capsaicin-sensitive C-fibers are capable of activating spinal circuits associated with pain. Using an identical experimental design, we found that intrathecal SP did not render peripheral injection of low-pH solutions (pH 3.5) noxious to naked mole-rats (Figure 7C). Hence, sensitization of spinal cord post-synaptic neurons is not sufficient to rescue acid nociception, further supporting our finding that naked mole-rat nociceptors are completely insensitive to acid.

## Discussion

In this paper we describe a constellation of pain related behaviors that are completely lacking in the African naked mole-rat (H. glaber). Pain behavior produced by two potent chemical algogens—capsaicin and protons—are completely absent in this species. We also show that the naked mole-rat lacks thermal hyperalgesia produced by algogenic chemicals and inflammation. Our detailed electrophysiological experiments reveal the neurobiological mechanisms that can account for the lack of specific pain-related behaviors in this species. We propose a model in which the extreme environment of the naked mole-rat, namely very high ambient CO_2_ concentrations, may have provided selective pressure resulting in the loss of specific pain behaviors in this species.

### Acid Insensitivity in the Naked Mole-Rat

Acid is a noxious stimulus for all vertebrate species so far studied. Thus, protons potently excite nociceptors in rats, mice, birds, frogs, and fish [8,21,43,44,48], and acid causes pain in humans [34,49] and pain-related behaviors in rodents, amphibians, and fish [8,43,50]. It is therefore very surprising that the naked mole-rat, a member of the rodent family, is behaviorally insensitive to protons (Figure 3B). We show that the lack of acid-induced behavior in the naked mole-rat can be explained by the fact that naked mole-rat nociceptors are not excited by proton concentrations that are maximally effective in other rodents (Figure 4D) [43,44]. It has been known for some time that mild tissue acidosis is a feature of inflammation in animals and humans [51,52] and this may directly contribute to inflammatory hyperalgesia [53]. We measured thermal and mechanical hyperalgesia in the CFA model and found that mechanical hyperalgesia occurs to a similar extent in both mice and naked mole-rats (Figure 3C), but thermal hyperalgesia is entirely absent in naked mole-rats (Figure 3D). Thus, mechanical hyperalgesia is unlikely to depend on acid-induced nociception. The absence of acid sensitivity in naked mole-rat nociceptors suggests that ion channels presumed to detect protons are absent or have altered function in this species. There is evidence that ion channels gated by protons may mediate acid nociception, thus members of the acid-sensing ion channel (ASICs) family, in particular ASIC3 [54], show sustained inward currents to low-pH solutions, and targeted mutation of the *ASIC3* gene reduces proton activation of nociceptors [43]. Another candidate acid sensor is the capsaicin-gated TRPV1 ion channel [1,11], which is both activated and sensitized by protons [12]. However, it has not yet been reported if mice lacking TRPV1 receptors have altered pain behavior following subcutaneous acid injection [41,55]. The naked mole-rat will prove to be a useful model to examine sequence variants in the genes encoding candidate ion channels that mediate acid transduction by nociceptors.

### Neurobiological Basis for Capsaicin Insensitivity in the Naked Mole-Rat

**Figure 8:**
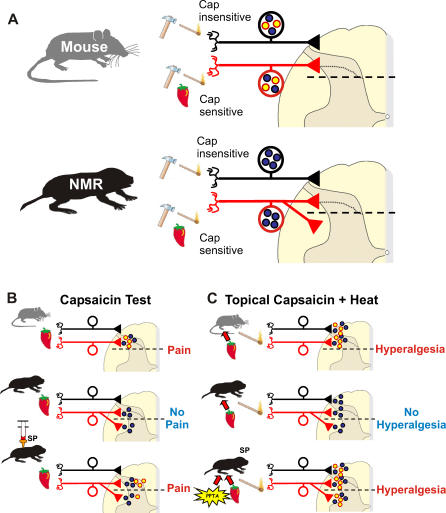
Model Showing the Patterns of C-fiber Innervation in the Spinal Cord of Mouse and Naked Mole-Rat (A) Both mouse and naked mole-rat have populations of capsaicin-insensitive and capsaicin-sensitive C-fiber nociceptors. Symbols (hammer, match, chilli pepper) indicate the type of stimuli (mechanical, thermal, chemical irritant) that can generate spiking activity in each population. In both species, capsaicin-insensitive and capsaicin-sensitive fibers terminate in the superficial lamina of the dorsal horn (above the dashed line in the spinal cord schematic). A major difference between species is that in the naked mole-rat, a substantial number of capsaicin-sensitive fibers also provide synaptic input to deep lamina. Another major difference is that C-fibers in naked mole-rats lack the neuropeptides SP and CGRP (yellow dots represent neuropeptides, blue dots represent glutamate). (B) Capsaicin test: Injection of capsaicin into the skin of the paw induces pain behaviors (licking) in mice but not naked mole-rats, except in naked mole-rats that have received an intrathecal infusion of SP. (C) Topical capsaicin + heat: Topical application of capsaicin induced thermal hyperalgesia in mice but not naked mole-rats, except in naked mole-rats that have received gene therapy with the *PPT* gene.

We showed that naked mole-rats are behaviorally insensitive to capsaicin. Birds and amphibians are also insensitive to capsaicin [10,56], and the avian *TRPV1* gene encodes a proton- and heat-sensitive channel that is not activated by capsaicin, which explains behavioral insensitivity in this species [10]. It thus appears that the capsaicin sensitivity of TRPV1 is an attribute gained in higher vertebrates during the course of evolution. In contrast, the naked mole-rat is behaviorally capsaicin-insensitive but has TRPV1 ion channels and nociceptors that are potently activated by capsaicin (Figure 4). We identified two populations of C-fiber nociceptors in the naked mole-rat: one is capsaicin sensitive and one is capsaicin insensitive (Figure 4 and Figure 8A). Noxious mechanical and thermal stimuli clearly lead to paw withdrawal, and chemical stimuli can produce paw licking and guarding behavior in the naked mole-rat (Figure 3). The question arises why activation of nociceptors with TRPV1 ion channels in these animals does not normally drive pain and hyperalgesia? To address this question, we measured the functional connectivity of capsaicin-sensitive nociceptors in the spinal cord dorsal horn. Whole-cell patch clamp recordings from spinal cord slices before and after capsaicin application indicated that capsaicin-sensitive C-fibers can robustly excite superficially located dorsal horn neurons both in the mouse and naked mole-rat (Figure 5). However, in the naked mole-rat, ∼50% of the deep dorsal horn neurons also receive direct synaptic input from capsaicin-sensitive fibers; this connectivity was much less pronounced in mouse slices (Figure 5). We also found that TRPV1-positive profiles in the deep dorsal horn were more prominent and numerous in the naked mole-rat compared to in the mouse (Figure 6). This finding provides an anatomical basis for the unusual functional connectivity that we observed in the naked mole-rat cord. It is well established that deep dorsal horn neurons, in general, usually receive multimodal sensory input with predominant direct input from mechanoreceptors, which signal innocuous stimulation [57]. Sensory information conveyed by C-fibers can, however, reach deep dorsal horn neurons, and some of this input may be direct monosynaptic input to the dendrites of deep dorsal horn neurons that extend into lamina II [58,59]. Interestingly, deep dorsal horn neurons that receive dendritic input from C-fibers express neurokinin-1 receptors, the receptor for SP that is absent from naked mole-rat nociceptors [18]. However, the number of deep dorsal horn neurons in the mouse that receive direct input via superficially located dendrites is likely to be low (Figure 5). Thus compared with mice, the naked mole-rat has an expanded functional connectivity between capsaicin-sensitive C-fibers and deep dorsal horn neurons. Noxious mechanical and heat stimuli activate C-fibers and lead to pain behaviors in the naked mole-rat (Figure 3A and 3B). However, exclusive excitation of capsaicin-sensitive C-fibers with thermal or mechanical stimuli probably do not lead to pain-related behavior. It appears that the novel connectivity of capsaicin-sensitive fibers fails to sufficiently activate circuits involved in the generation of pain behavior. We postulate that capsaicin-sensitive C-fibers connect to a neuronal network in the naked mole-rat spinal cord, the integrated output of which is insufficient to generate pain-related behavior. Capsaicin-insensitive fibers in the naked mole-rat do activate circuits involved in the generation of pain (Figure 3A and 3B), and our data suggest that these are the only C-fibers that normally mediate acute nociception and mechanical hyperalgesia in this species.

It should be noted that the naked mole-rat is not the only species in which sensory neurons are found that respond to capsaicin but are not activated by heat [60–62]. It is, however, possible that our heat stimulus did not activate a small population of C-fibers with very high noxious heat thresholds. However, it has been noted in other rodent species that essentially all noxious heat-sensitive neurons respond to capsaicin [63,64]. We found that only 50% of naked mole-rat C_MH_ neurons were excited by capsaicin (Figure 4C). Heat sensitivity is not necessarily inseparable from capsaicin sensitivity, because C-fibers in TRPV1-null mutant mice apparently have normal noxious heat sensitivity but are capsaicin insensitive [65].

### No Thermal Hyperalgesia in the Naked Mole-Rat

Thermal hyperalgesia following inflammation is thought to be explained, in part, by the sensitization of primary afferent nociceptors to heat [3]. One important chemical mediator in this process is NGF, which is up-regulated in inflamed tissues and can directly sensitize nociceptors [36,37,42]. TRPV1 has been proposed to be a crucial integrator of inflammatory signals to mediate thermal hyperalgesia [1,11]. We show that naked mole-rats have functional TRPV1 receptors that can be gated by capsaicin (Figure 4), yet this species shows no thermal hyperalgesia. Nevertheless, the fact that TRPV1-positive C-fibers normally fail to drive circuits leading to pain behavior (Figure 8A) may account for the lack of thermal hypersensitivity in this species following inflammation and NGF.

### Role of Sensory Neuropeptides in Naked Mole-Rat Pain Biology

We initially found that the neuropeptides SP and CGRP are both missing from naked mole-rat nociceptors innervating the skin [18]. Mice lacking SP and CGRP show reduced pain-related behavior [19,20], and mice with a mutation in the *PPT* gene that encodes SP are also less sensitive to capsaicin [19]. However, the deficits in pain behavior described here are more extensive than can be explained simply by the absence of these neuropeptides. Nevertheless, we decided to ask whether reintroduction of the SP into the spinal cord or DRG neurons in naked mole-rats could “normalize” pain behaviors in this species. We did find that intrathecal administration of SP in the naked mole-rat could produce thermal hyperalgesia as has been described in other rodents, albeit at higher doses. Consistent with this, we were able to detect the neurokinin-1 (NK1) receptor immunoreactivity in naked mole-rat superficial spinal cord neurons (Figure S4). We used a neurotropic virus to reintroduce *PPT* gene expression into naked mole-rat sensory neurons and found that capsaicin could now induce thermal hyperalgesia in the treated mole-rats (Figure 7B). Finally, intrathecal administration of SP rendered capsaicin injection painful to naked mole-rats. It is not clear if this finding is of physiological significance for this species, but it is conceivable that at some point in the long and complex life history of the naked mole-rat, environmental factors might induce neuropeptide production by DRG neurons.

Our data on the acid insensitivity and altered connectivity of naked mole-rat C-fibers suggest that the lack of neuropeptides is not the sole cause of altered pain behavior in this species. We propose a model that can account for the “rescue” effects of SP in the naked mole-rat. We suggest that there is a balance between C-fiber synaptic drive to superficial and deep dorsal horn neurons, so that pain or thermal hyperalgesia result only when the activation of superficial dorsal horn neurons predominates (Figure 8B and 8C). Thus glutamatergic input driven by thermal and mechanical stimuli is sufficient to drive pain behavior, because such stimuli drive both capsaicin-sensitive and -insensitive C-fibers (net input to superficial laminae ≫ deeper laminae). Intrathecal SP may selectively sensitize superficially located neurons to C-fiber input from both groups of C-fibers, so that capsaicin can produce thermal hyperalgesia (Figure 8B). After reintroducing the gene encoding SP into both types of C-fiber, capsaicin stimulation should lead to SP release both superficially and deep. Under this condition, thermal hyperalgesia results as natural stimuli lead to more activation in superficial layers as more C_MH_ fibers terminate here (Figure 8C). This is a speculative model that can potentially account for our observations but its validity must be tested with further experiments.

### Why No Pain in the African Naked Mole-Rat?

We show that nociceptors in the naked mole-rat have acquired specific molecular changes affecting their function and central connectivity that serve to minimize chemical nociception and abolish thermal hyperalgesia. What kind of selection pressure has led to the evolution of such an unusual nociceptive system? One explanation may lie in the social organization of this subterranean mammal. In the wild, hundreds of naked mole-rats live in cramped, poorly ventilated chambers, where O_2_ levels are low and CO_2_ levels are high [66]. Consequently, naked mole-rats have adapted to survive in low-O_2_ concentrations [67]. High levels of CO_2_ are a noxious stimulus [68] and can stimulate C-fibers by producing tissue acidosis [44]; indeed even moderately high levels of CO_2_ can cause pulmonary edema presumably by activating peptidergic afferents in the lung [69]. We postulate that high ambient CO_2_ levels in the burrows of a naked mole-rat ancestor might have produced selective pressure to abolish acid activation of nociceptors and the consequent release of neuropeptides both centrally and peripherally. The inflammatory pain insensitivity in these animals may therefore be a by-product of adaptations essential for species success. Further molecular and cellular studies on the nociceptive system in these animals may provide significant insights into components that are necessary for the transduction of painful stimuli in other mammals including humans.

## Materials and Methods

### Behavioral experiments.

Withdrawal times to thermal stimuli were measured by using a radiant heat source focused on the hind-paw of lightly anaesthetized animals (pentobarbital 35 mg/kg, intraperitoneal [ip] ). Licking times after administration of algogenic chemicals were measured using a stopwatch to the nearest 0.1 s. Mechanical thresholds were determined using a series of calibrated von Frey hairs to determine the minimum force required to elicit paw withdrawal before and after CFA. The mice used in behavioral experiments were male Swiss Webster.

For intrathecal injections, animals were lightly anesthetized with pentobarbital (35 mg/kg in physiological saline, ip). SP (1, 10, or 100 μM, 20 μl in physiological saline) was injected intrathecally between vertebrae L4 and L5. For the intrathecal (IT) plus Cap test, animals were briefly (∼ 1 min) anesthetized with metophane for the IT injection. Five to 10 m later, the capsaicin test was administered as described above. The University of Illinois at Chicago Institutional Animal Care and Use Committee and German National Oversight bodies approved the animal protocols used here.

### Electrophysiology and Ca^2+^ imaging.

Animals were decapitated, and the skin from the area innervated by the saphenous nerve was removed with the nerve intact. After dissection, the preparation was placed in an organ bath with the corium side of the skin facing up to ensure efficient oxygenation. The preparation was superfused with an oxygen-saturated modified interstitial fluid solution containing (mM): 123 NaCl, 3.5 KCl, 0.7 MgSO4, 1.7 NaH2PO4, 2.0 CaCl2, 9.5 sodium gluconate, 5.5 glucose, 7.5 sucrose, and 10 HEPES, adjusted to pH 7.4 ± 0.05, temperature, 32 ± 0.5 °C. The saphenous nerve was desheathed, and individual filaments were teased away, enabling extracellular recordings to be made from functionally identified single fibers [24,32]. A template of the spike under study was saved on the oscilloscope (Tektronix TDS 200) and evoked spikes were visually monitored to make sure that no other active spikes would be mistaken for the one under study. All data were collected and saved to disk using Chart software for the Powerlab system running on a PC (ADInstruments). For each single unit, the data were analyzed off-line using the spike histogram extension of Chart software. This software allows calculation of histograms of spikes discriminated on the basis of a constant height and width. For the application of capsaicin and heated buffer solution, a metal ring (diameter = 8 mm) was used to isolate the receptive field and application of 100 μl of capsaicin in buffer solution, or heated buffer solution was applied into the ring. The temperature at the corium surface of the skin was measured with a thermocouple and reached >45 °C very shortly after application of the heated solution.

Recordings of the ventral root potential in naked mole-rats were also carried out as previously described [47]. Spinal cords were removed from 1-y-old naked mole-rats and neonatal mice (P5-P9) and were hemisected down the midline, placed in a Perspex recording chamber, and superfused with oxygenated modified Krebs solution. This preparation does not appear to be viable in older mice probably because of increasing myelin content of the cord. Only one hemisected cord was used per animal. Direct current recordings were made after a 2-h recovery period with a close-fitting glass suction electrode attached to the L5 ventral root. The L5 dorsal root was stimulated via a glass suction electrode at current sufficient to activate C- fibers (500 μA, 500 μs).

We used standard Fura-2–based ratiometric calcium imaging techniques to record responses to application of capsaicin solutions in cultured DRG neurons from the naked mole-rat and mouse. An inverted microscope (Zeiss Axiovert200) equipped with TILL photonics imaging system, including the polychrome V, a CCD camera, and the imaging software TILLvisION was used for cell imaging. Paired images (340 and 380 nm excitation, 510 nm emission) were collected every 4 s.

### Slice preparation.

Laboratory mice and naked mole-rats were anaesthetized and decapitated. The spinal column was quickly removed and placed in an ice-cold dissection solution with a reduced calcium concentration consisting of (mM): 125 NaCl, 4 KCl, 10 glucose, 1.25 NaH_2_PO_4_, 25 NaHCO_3_, 0.1 CaCl_2_, and 3.0 MgCl_2_. The spinal cords (SCs) were removed, embedded in agarose (2.5%, SeaPlaque agarose, CAMBREX), and transversal slices (180 μm) were prepared by vibratome cutting. Slices were maintained at least 1 h before recording at room temperature. Whole-cell patch clamp recordings of mEPSCs were performed similar to the method of Baccei and colleagues [45]. Briefly, mEPSCs were recorded from superficial and deep dorsal horn neurons. Cell locations were reconstructed from photographic images of the slices with the recording pipette in place. AMPA-mediated mEPSCs were isolated pharmacologically by blocking glycinergic and GABAergic input as well as NMDA receptor-mediated currents (strychnine, 1.0 μM; picrotoxin, 100 μM; APV, 100 μM). Action potential–dependent neurotransmitter release was blocked by TTX (1 μM). All experiments were performed at room temperature. During recordings, slices were perfused at a rate of 2 ml/min with bath solution of (in mM): 125 NaCl, 4 KCl, 10 glucose, 1.25 NaH_2_PO_4_, 25 NaHCO_3_, 2.0 CaCl_2_, and 1.0 MgCl_2_. The patch pipette solution contained (in mM): 120 CsCl, 4 NaCl, 5 glucose, 5 ethylene glycol-bis(β-aminoethyl ether) N,N,N′,N′-tretraacetic acid (EGTA), 10 N-2-hydroxyethylpiperzine-N′-2-ethanesulfonic acid (HEPES), 0.5 CaCl_2_ and 4 MgCl_2_ (pH 7.3). During recordings at a holding potential of −70 mV, the effective access resistance was in the range of 10 to 40 MOhm and was checked throughout the experiment by using a short depolarizing pulse. Recordings were included in the analysis only if the access resistance was less than 40 MOhm and did not change more than 20% during the experiment. Capsaicin (10 μM) was applied by a superfusion pipette (diameter 150–200 μm) for 100 s. Recordings were made using an EPC-9 (HEKA Electronics). Signals were sampled at a rate of 10 kHz and analysed using WinTida 5.0 (HEKA Electronics). Postsynaptic currents were filtered at 3 kHz and analyzed off-line by MiniAnalysis (Synaptosoft).

### Cell culture and immunohistochemistry.

DRG neurons from naked mole-rats were placed in culture using essentially identical methods to those described previously by us for mouse neurons [30,70]. Cells were usually cultured overnight before calcium imaging experiments or immunocytochemistry. We have used the following antibodies: anti-neurofilament H (Affiniti, mouse monoclonal Clone RT-97), anti-TrkA (rabbit polyclonal a kind gift from L.F. Reichardt, University of California San Francisco), anti-substance P receptor (NK1 receptor, rabbit polyclonal, Sigma), and anti-TRPV1 (Calbiochem, rabbit polyclonal). We used fluorescently labeled IB4 (Alexa 488 Molecular probes) to label cultured mouse and naked mole-rat sensory cells as previously described [30].

### Transgenic herpes virus.

Replication-conditional, recombinant herpes viruses were created using the KOS strain of herpes simplex virus, type I (HSV). The cDNA for rat PPT, generously provided by J.E. Krause (Washington University School of Medicine), supplied in plasmid pG1β-PPT [71] was blunt cloned as a 512-bp BamH I fragment into the Not I site of the HSV shuttle plasmid pTKV2, which contains the hCMV immediate-early promoter/enhancer, an SV40 splice site, and the SV40 polyadenylation sequence derived from pCMVβ [72]. This shuttle vector targets the HSV thymidine kinase gene, causing insertional inactivation of the thymidine kinase gene. Inactivation of thymidine kinase prevents viral replication in neurons, but allows replication in Vero cells and in skin cells. Inserts containing the β-PPT cDNA were selected and characterized by restriction mapping. For generation of the sense and antisense viruses containing the PPT cDNA, the linearized shuttle plasmids were individually transfected into Vero cells with Pac I-digested viral DNA that contained a similar lacZ expression cassette flanked with Pac I restriction sites (virus PZ, method of [73]). The resultant recombinants were purified to homogeneity by three rounds of limiting dilution and the expected insertion into the virus confirmed by Southern blotting. Virus stocks were prepared using Vero cells, concentrated by centrifugation and stored in MEM containing 10% cosmic calf serum (HyClone), 5 mM HEPES, pH 7.4 and 10% glycerol at −80°.

## Supporting Information

Figure S1 Axon Diameter Size Distribution of Myelinated Fibers from the Saphenous Nerve from Naked Mole-Rat Compared to the Mouse The mean diameter of myelinated fibers in the mouse was significantly larger than that found in the naked mole-rat, *p* < 0.0001, unpaired *t*-test, although this difference was small.
(16 KB PDF)

Figure S2 NGF Stimulates Neurite Outgrowth in Cultivated Naked Mole-Rat Sensory Neurons Fluorescent photomicrographs are shown of naked mole-rat sensory neurons in culture double stained for neurofilament heavy chain (NF200 green) and the NGF receptor trk A (red). Note that in the absence of NGF (top micrographs), naked mole-rat sensory neurons elaborate very few neurites. In the presence of 500 ng/ml of NGF, sensory neurons elaborated extensive neurites and the same cells were positive for the trk A receptor (middle and bottom micrographs). The scale bar is 50 μm.
(151 KB PDF)

Figure S3 Ventral Root Reflexes Measured in Mouse and Naked Mole-Rat In Vitro Spinal Cord Preparations (A) Representative ventral root potentials (VRPs) evoked by single-shock C-fiber strength electrical stimulation of the corresponding dorsal root obtained from a hemisected spinal cord from neonatal mouse and adult naked mole-rat. Note that the large and very long-lasting VRP in the mouse is not observed to the same extent in the naked mole-rat. The amplitude of the second long-lasting component is considerably smaller than that found in the mouse.
(B) Quantification of the amplitude of the potential in mouse and naked mole-rat. No difference was found in the putative A-fiber component, but the C-fiber component was reduced by over 70% in the naked mole-rat compared to the mouse.
(36 KB PDF)

Figure S4 NK-1 Receptor Staining in the Spinal Cord of the Mouse and Naked Mole-Rat Sections were stained with a primary anti-rat NK-1 antibody that was detected with a secondary antibody conjugated to Cy3 (red).
(A) mouse and (B) naked mole-rat. Staining in the mouse is primarily limited to the lamina I region whereas in the naked mole-rat, there is staining deeper into lamina II. Scale bar represents 100 μm.
(282 KB PDF)

Table S1 The Decay Time Constant of the mEPSC Was Calculated with a Monoexponential Fit Numbers in brackets are the total number of recorded neurons. The mean frequency is calculated for each neuron before the application of capsaicin.
(27 KB DOC)

## Abbreviations

AM: A-fiber mechanonociceptor
ASIC: acid-sensitive ion channel
C_-M_: C-fiber mechanonociceptor
C_-MH_: C-fiber mechanoheat nociceptor
CFA: complete Freund's adjuvant
CGRP: calcitonin gene-related peptide
CNS: central nervous system
DRG: dorsal root ganglia
HSV: herpes simplex virus
IB4: isolectin B4
IT: intrathecal
mEPSC: miniature excitatory post-synaptic current
NGF: nerve growth factor
PPT: preprotachykinin
SP: substance P
TRPV1: transient receptor potential vanilloid channel-1
TTX: tetrodotoxin
