# Supplementary figures and images for: Selective Inflammatory Pain Insensitivity in the African Naked Mole-Rat (Heterocephalus glaber)
Source: PLoS Biol. 2008 Jan 29;6(1):e13. doi: 10.1371/journal.pbio.0060013 (PMC2214810; doi:10.1371/journal.pbio.0060013)

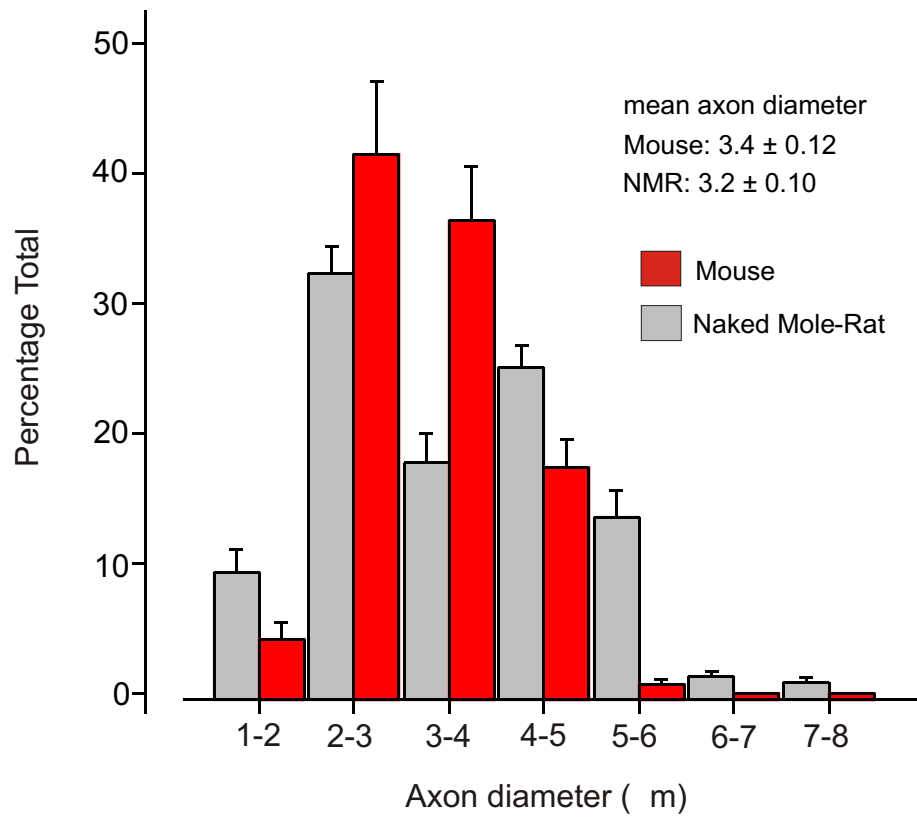

Park et al Supplementary Figure 1

Supplement: Figure S1 — The mean diameter of myelinated fibers in the mouse was significantly larger than that found in the naked mole-rat, p < 0.0001, unpaired t-test, although this difference was small. (16 KB PDF) [file pbio.0060013.sg001.pdf]

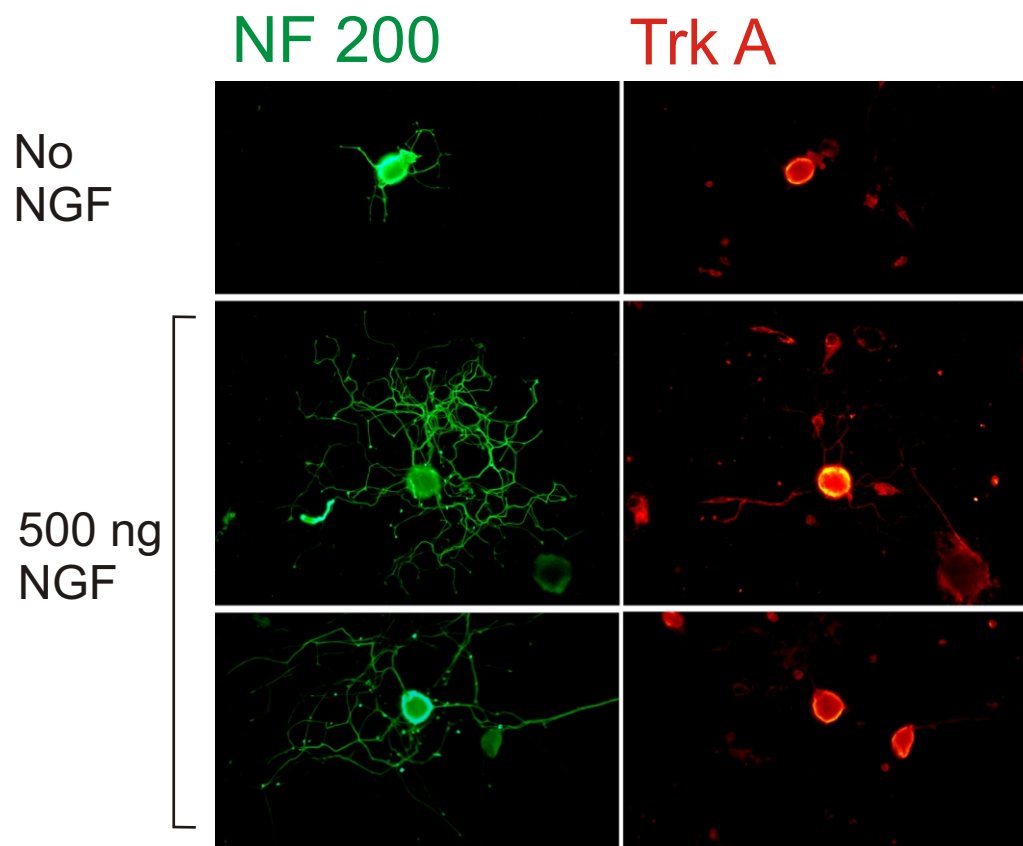

Park et al Supplementary Figure 2

Supplement: Figure S2 — Fluorescent photomicrographs are shown of naked mole-rat sensory neurons in culture double stained for neurofilament heavy chain (NF200 green) and the NGF receptor trk A (red). Note that in the absence of NGF (top micrographs), naked mole-rat sensory neurons elaborate very few neurites. In the presence of 500 ng/ml of NGF, sensory neurons elaborated extensive neurites and the same cells were positive for the trk A receptor (middle and bottom micrographs). The scale bar is 50 μm. (151 KB PDF) [file pbio.0060013.sg002.pdf]

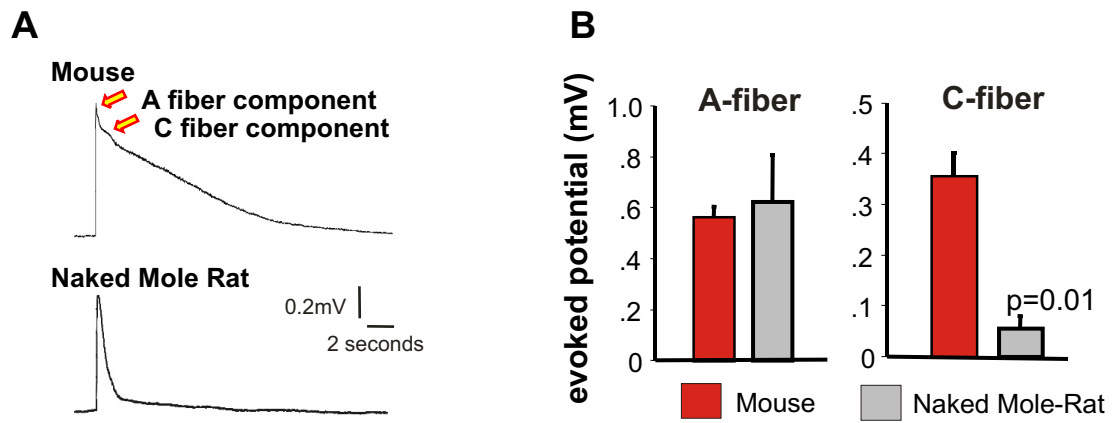

Park et al Supplementary Figure 3

Supplement: Figure S3 — (A) Representative ventral root potentials (VRPs) evoked by single-shock C-fiber strength electrical stimulation of the corresponding dorsal root obtained from a hemisected spinal cord from neonatal mouse and adult naked mole-rat. Note that the large and very long-lasting VRP in the mouse is not observed to the same extent in the naked mole-rat. The amplitude of the second long-lasting component is considerably smaller than that found in the mouse. (B) Quantification of the amplitude of the potential in mouse and naked mole-rat. No difference was found in the putative A-fiber component, but the C-fiber component was reduced by over 70% in the naked mole-rat compared to the mouse. (36 KB PDF) [file pbio.0060013.sg003.pdf]

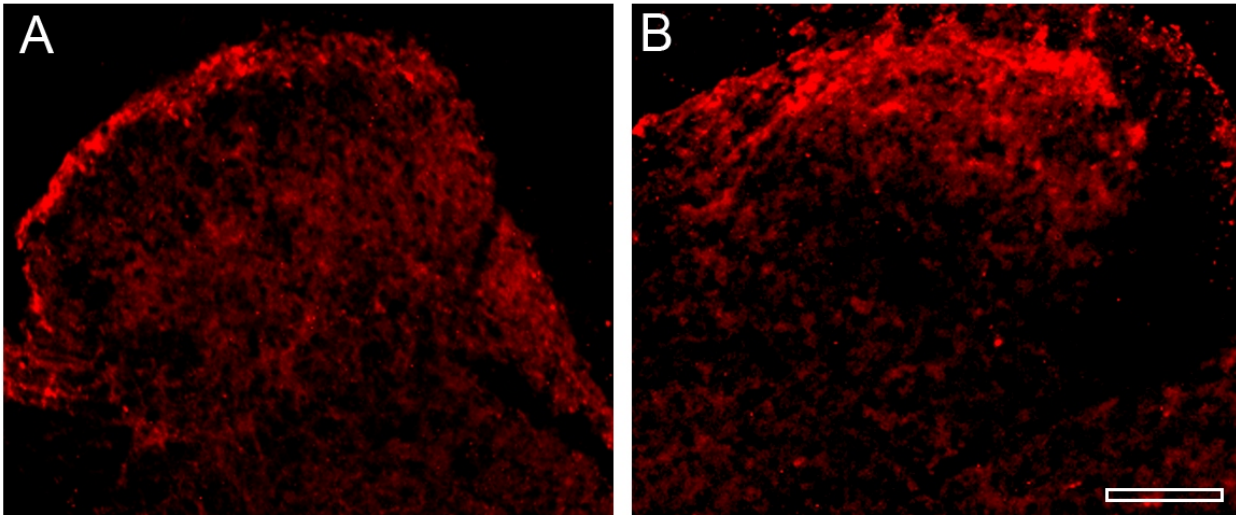

Park et al Supplementary Figure 4

Supplement: Figure S4 — Sections were stained with a primary anti-rat NK-1 antibody that was detected with a secondary antibody conjugated to Cy3 (red). (A) mouse and (B) naked mole-rat. Staining in the mouse is primarily limited to the lamina I region whereas in the naked mole-rat, there is staining deeper into lamina II. Scale bar represents 100 μm. (282 KB PDF) [file pbio.0060013.sg004.pdf]
