# Supplementary material for: Selective Inflammatory Pain Insensitivity in the African Naked Mole-Rat (Heterocephalus glaber)
Source: PLoS Biol. 2008 Jan 29;6(1):e13. doi: 10.1371/journal.pbio.0060013 (PMC2214810; doi:10.1371/journal.pbio.0060013)
Supplement: Table S1 — Numbers in brackets are the total number of recorded neurons. The mean frequency is calculated for each neuron before the application of capsaicin. (27 KB DOC) [file pbio.0060013.st001.doc]

**Supplementary Table 1: mEPSCs properties**

|  | **Mouse** | **NMR** |
| --- | --- | --- |
| Amplitude (pA) | 21.98 ± 1.08 (52) | 24.86 ± 1.67 (19) |
| Decay time (msec) | 2.13 ± 0.11 (51) | 1.90 ± 0.13 (19) |
| Frequency (events/sec) | 0.299 ± 0.062 (63) | 0.188 ± 0.053 (21) |
